# Synthesizing Existing CSMA and TDMA Based MAC Protocols for VANETs

**DOI:** 10.3390/s17020338

**Published:** 2017-02-10

**Authors:** Jiawei Huang, Qi Li, Shaohua Zhong, Lianhai Liu, Ping Zhong, Jianxin Wang, Jin Ye

**Affiliations:** 1 School of Information Science and Engineering, Central South University, Changsha 410083, China; jiaweihuang@csu.edu.cn (J.H.); liqi_15@csu.edu.cn (Q.L.); zhongshaohua@csu.edu.cn (S.Z.); llh@guet.edu.cn (L.L.); jxwang@csu.edu.cn (J.W.); 2 School of Computer, Electronics and Information, Guangxi University, Nanning 530004, China; yejin@gxu.edu.cn

**Keywords:** VANET, MAC, CSMA, TDMA

## Abstract

Many Carrier Sense Multiple Access (CSMA) and Time Division Multiple Access (TDMA) based medium access control (MAC) protocols for vehicular ad hoc networks (VANETs) have been proposed recently. Contrary to the common perception that they are competitors, we argue that the underlying strategies used in these MAC protocols are complementary. Based on this insight, we design CTMAC, a MAC protocol that synthesizes existing strategies; namely, random accessing channel (used in CSMA-style protocols) and arbitral reserving channel (used in TDMA-based protocols). CTMAC swiftly changes its strategy according to the vehicle density, and its performance is better than the state-of-the-art protocols. We evaluate CTMAC using at-scale simulations. Our results show that CTMAC reduces the channel completion time and increases the network goodput by 45% for a wide range of application workloads and network settings.

## 1. Introduction

During the last decade, vehicular ad hoc networks (VANETs) have attracted significant interest from both academia and industry. As self-organized networks built up from moving vehicle nodes, VANET provides both inter-vehicle (V2V) communications between vehicles and vehicle-to-roadside (V2R) communications between vehicles and roadside units (RSUs) [1,2]. The main characteristics of VANETs are high mobility and ample energy, which respectively lead to highly dynamic network topology and large transmission range (i.e., 500 m). In VANETs, since the nodes share a common wireless channel by using the same radio frequency, an inappropriate use of the wireless channel easily results in transmission collisions and bandwidth wasting.

Due to the high mobility of vehicles, it is challenging to design an efficient medium access control (MAC) protocol with low delay and high reliability. Several research projects, such as Fleet-Net [3], PReVENT [4], SAFESPOT [5], C2C-CC [6], and CarTALK [7] are dealing with vehicular communications. Existing proposals have developed CSMA (Carrier Sense Multiple Access)- and TDMA (Time Division Multiple Access)-based MAC protocols to tackle this problem [8]. However, all of these protocols have their own strengths and weaknesses: CSMA-based MAC protocols perform well for low vehicle density, but bring about high collision in the presence of vehicle crowding due to the random backoff method, while TDMA-based MAC protocols provide deterministic delay bounds by resource reservations, but are sensitive to underlying mobility and topology changes.

When the vehicles move with high speed, the network topology varies rapidly, leading to ever-changing scenarios. We observe that each strategy of MAC protocols has its own role. It works best for some scenarios and encounters problems otherwise. Unfortunately, existing protocols only use one of the CSMA and TDMA strategies and try to make it work under all scenarios. In this paper, we argue that both CSMA and TDMA strategies should be used together, as they nicely complement each other. Specifically, CSMA strategy is suitable for sparse network scenarios, because the underlying random accessing strategy achieves high utilization with low channel contention. Conversely, arbitration of the TDMA strategy can provide the bounded delay (even for large vehicle crowding), but it may waste channel resources—especially at low loads when the vehicle density is small.

To demonstrate the benefits of using these strategies together, we design CTMAC—a MAC protocol that synthesizes the random accessing channel (used in CSMA-style protocols) and the arbitral reserving channel (used in TDMA-based protocols). CTMAC determines the number of backoff time slots according to the vehicle density, seamlessly switching the channel accessing strategy between CSMA and TDMA. For low vehicle density, CTMAC employs the random backoff to achieve high channel utilization, while CTMAC uses the fixed backoff to reserve channel and thus guarantees the bounded delay, even in the case of large vehicle crowding. Our contributions are as follows:

- We provide an extensive study to experimentally and theoretically highlight the strengths and weaknesses of existing VANET MAC protocols. We reveal the impact of vehicle density on network goodput, and demonstrate why the synthesis of CSMA and TDMA is more effective under varying vehicle density.
- We design a MAC protocol that synthesizes the strategies of random accessing channel and arbitral reserving channel. Our CTMAC protocol swiftly changes its strategy by only adjusting the number of backoff time slots, which requires little modification of MAC protocols and can be easily deployed.
- We evaluate our design on NS2 simulations. The results demonstrate that—as with CTMAC—we are able to broadly improve the network goodput of state-of-the-art MAC protocols by 45%. We also show that CTMAC outperforms other protocols in terms of delay.

The remainder of this paper is structured as follows. In Section 1, we describe our design rationale. The details of CTMAC are presented in Section 2. In Section 3, we report the results of performance evaluation. In Section 4, we demonstrate existing approaches and discuss their pros and cons. Finally, we conclude the paper in Section 5.

## 2. Motivation

In this section, we present empirical studies to show the impact of varying vehicle density, as it is very common in VANET. Then, we theoretically demonstrate that high network goodput is obtained by synthesizing the CSMA- and TDMA-based MAC Protocols.

### 2.1. Impact of Varying Vehicle Density

In VANET, the high mobility of vehicles leads to frequently changing network topology. For a given vehicle node, when the other vehicles move in and out of its transmission range with high speed, the vehicle density around the given vehicle node varies quickly, having a great impact on the performance of MAC protocols.

Here, we firstly use NS2 simulation [9] to show the impact of vehicle density. All vehicles are uniformly deployed. Since IEEE 802.11p [10] is the MAC-layer standard in VANETs, we chose it as the CSMA protocol and compare it with VeMAC [11] protocol, which is an enhanced TDMA-based protocol designed for VANETs. Table 1 shows the road traffic parameters and MAC protocol settings that are used for both the simulations and the following analysis.

**Figure 1:**
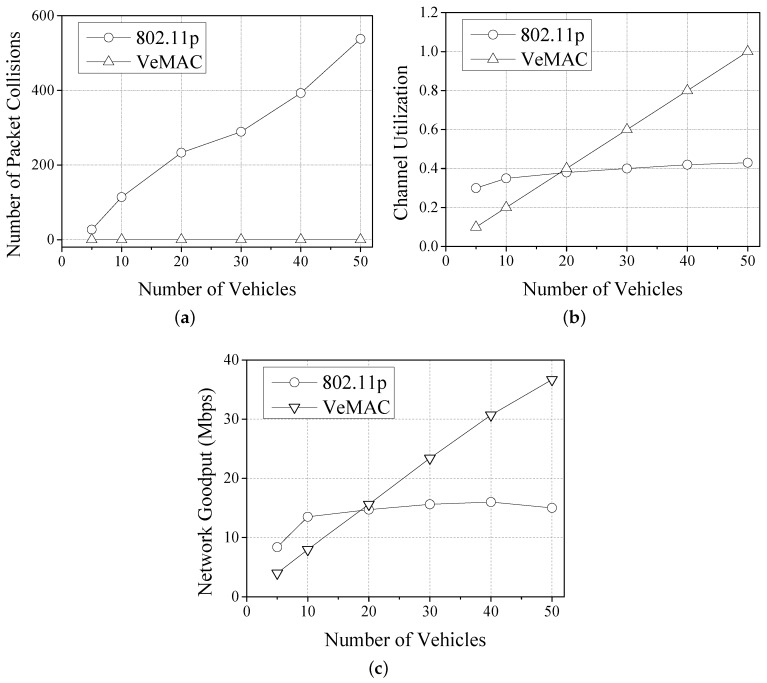
Comparison of IEEE 802.11p and VeMAC with different numbers of vehicles. (**a**) Packet collision; (**b**) Channel utilization; (**c**) Network goodput.

We compare the number of packet collisions, channel utilization, and network goodput of IEEE 802.11p and VeMAC protocols under a varying number of vehicles. The number of vehicles was increased from 5 to 50. Figure 1a shows the number of packet collisions against the number of vehicles. When the number of vehicles is only 5, the packet collision is slight for 802.11p; yet, the collision occurs frequently with the increasing of number of vehicles due to its random accessing channel. By contrast, since it coordinates the time slots of each vehicle, TDMA experiences no packet collisions throughout the test. Figure 1b shows the channel utilization of both protocols. Since the number of slots in TDMA Frame *N* is fixed, if the number of vehicles is less than *N*, the channel utilization of TDMA is degraded. Therefore, for a small number of vehicles, 802.11p obtains much higher channel utilization than TDMA. However, 802.11p is not able to provide high channel utilization for a large number of vehicles, because its heavy packet collision leads to large backoff time and channel wastage. Finally, we compare the network goodput as shown in Figure 1c. It is obvious that 802.11p achieves higher network goodput than TDMA for a small number of vehicles, while TDMA obtains higher network goodput for a large number of vehicles.

### 2.2. Model Analysis

In this subsection, we use the mathematical model to demonstrate why higher network goodput is obtained by synthesizing the CSMA and TDMA.

We model the backoff procedure of the IEEE 802.11p protocol as a *p*-persistent CSMA. We note that the backoff interval of the *p*-persistent CSMA is sampled from a geometric distribution with transmission probability *p*, which is different from the binary exponential backoff in IEEE 802.11p. The reason for this is that the *p*-persistent CSMA provides a very close approximation to the IEEE 802.11 protocol, and the memoryless backoff algorithm makes it suitable for mathematical analysis.

**Figure 2:**
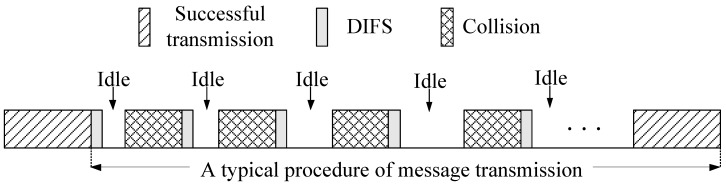
A typical procedure of message transmission.

Figure 2 shows the procedure before each successful message transmission. Packet collisions and idle periods may occur before a successful transmission. The packet collision happens when at least two vehicles transmit at the same time slot. The idle period is a time interval (expressed in number of time slots) in which the transmission medium remains free of any transmission when all vehicles are in their backoff time.

Assuming that *n* contending vehicles may send the data at the same time, the probability of successful transmission $P_{s}$ and collision probability $P_{c}$ are

(1) $$P_{s}=\frac{np{\left(1-p\right)}^{n-1}}{1-{\left(1-p\right)}^{n}},$$

(2) $$P_{c}=\frac{1-{\left(1-p\right)}^{n}-np{\left(1-p\right)}^{n-1}}{1-{\left(1-p\right)}^{n}},$$

where the transmission probability *p* can be calculated with the minimum value $CW_{min}$ of contention window as

(3) $$p=\frac{2}{{CW}_{min}+1}.$$

From Equations (1) and (2), the expected value $E[N_{c}]$ of number of collisions before a successful transmission can be calculated as

(4) $$E\left[N_{c}\right]=\frac{P_{c}}{P_{s}}=\frac{1-{\left(1-p\right)}^{n}}{np{\left(1-p\right)}^{n-1}}-1.$$

For each transmission collision, the collision time $T_{c}$ includes the packet’s transmission time σs and the DIFS time σD. The packet size *s* is expressed as time slots. For example, it corresponds to the transmission time of time 32 slots, giving the 1 Mbps wireless transmission rate. Therefore, the total collision time $T_{col}$ before a successful transmission is

(5) $$T_{col}=E\left[N_{c}\right]T_{c}=\sigma \left[\frac{1-{\left(1-p\right)}^{n}}{np{\left(1-p\right)}^{n-1}}-1\right]\left(s+D\right).$$

As shown in Figure 2 , since a collision is just between two idle periods, the expected number $E[N_{i}]$ of idle period is

(6) $$\begin{array}{cc} E[N_{i}] & =E[N_{c}]+1\\ & =\frac{1-{\left(1-p\right)}^{n}}{np{\left(1-p\right)}^{n-1}}. \end{array}$$

The number of time slots $T_{i}$ in each idle period is determined by the transmission probability *p* and the number of contending vehicles *n*. The expected value of $E[T_{i}]$ is

(7) $$\begin{array}{cc} E[T_{i}] & =\sigma \left[1-{\left(1-p\right)}^{n}\right]\sum_{i=0}^{\propto }i{\left(1-p\right)}^{ni}\\ & =\frac{\sigma {\left(1-p\right)}^{n}}{1-{\left(1-p\right)}^{n}}. \end{array}$$

Thus, the total collision time $T_{idle}$ before a successful transmission can be calculated as

(8) $$T_{idle}=E\left[N_{i}\right]E\left[T_{i}\right]=\frac{\sigma (1-p)}{np}.$$

The successful transmission time $T_{trans}$ is

(9) $$T_{trans}=\sigma \left(s+D\right).$$

Since the transmission time $T_{total}$ is composed of the collision time $T_{col}$, idle time $T_{idle}$, and successful transmission time $T_{trans}$, we have

(10) $$\begin{array}{cc} T_{total} & =T_{col}+T_{idle}+T_{trans}\\ & =\frac{\sigma [s+D-\left(s+D-1\right){\left(1-p\right)}^{n}]}{np{\left(1-p\right)}^{n-1}}. \end{array}$$

Therefore, the network goodput of IEEE 802.11p protocol can be expressed as

(11) $$\begin{array}{cc} G_{CSMA} & =\frac{s}{T_{total}}\\ & =\frac{np{\left(1-p\right)}^{n-1}s}{\sigma [s+D-\left(s+D-1\right){\left(1-p\right)}^{n}]}. \end{array}$$

A TDMA-based MAC protocol is used to enable multiple vehicles to transmit on the same frequency channel. It divides the signal into different time frames. Each time frame is divided into several time slots, where each vehicle is assigned to a time slot for collision-free transmission. To accommodate more vehicles, the number of time slots *N* in each frame is usually fixed as a large number (i.e., 50 as default). Given the number of vehicles *n* and the packet size *s*, the network goodput can be calculated as

(12) $$G_{TDMA}=\frac{ns}{N\sigma }.$$

**Figure 3:**
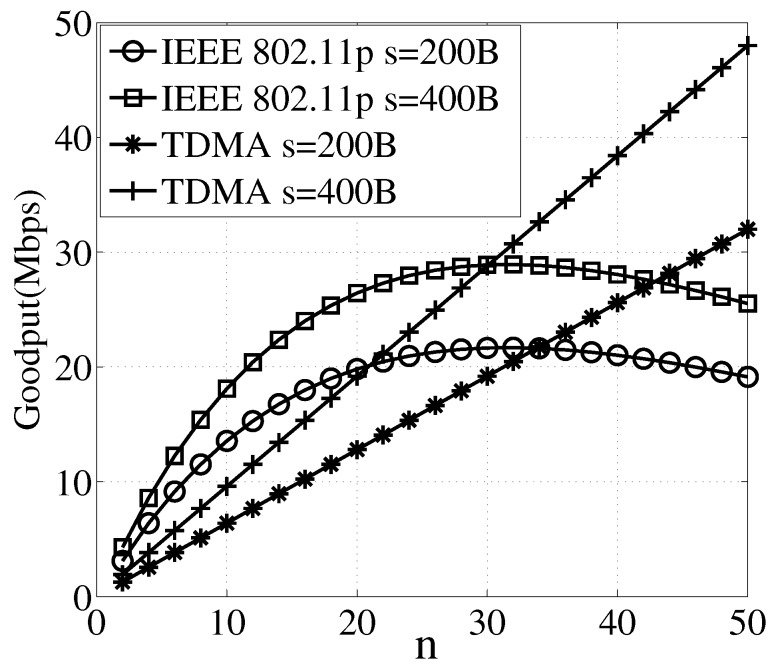
Network goodput with different number of vehicles.

Next, we show why higher network goodput is obtained by synthesizing the CSMA and TDMA. We change the number of vehicles from 5 to 50, and show the network goodput in Figure 3. The theoretical result validates the simulation result with the increasing of number of vehicles *n* under different packet sizes *s*. When the number of vehicles is small, the 802.11p protocol achieves higher network goodput, but with the increasing of number of vehicles, the TDMA protocol performs better.

Our observation leads us to conclude that both CSMA- and TDMA-based protocols are sensitive to the number of vehicles, which suggests that adopting a fixed strategy is not an optimal solution under dynamic network traffic scenarios. These conclusions motivated us to investigate a novel approach synthesizing the CSMA- and TDMA-based MAC protocols. In the rest of this paper, we present our design in detail.

## 3. Protocol Design

In this section, we describe the design detail of CTMAC, including how to determine the number of backoff time slots according to the vehicle density, and thus switch the channel accessing strategy seamlessly between CSMA and TDMA.

### 3.1. Synthesizing CSMA and TDMA

**Figure 4:**
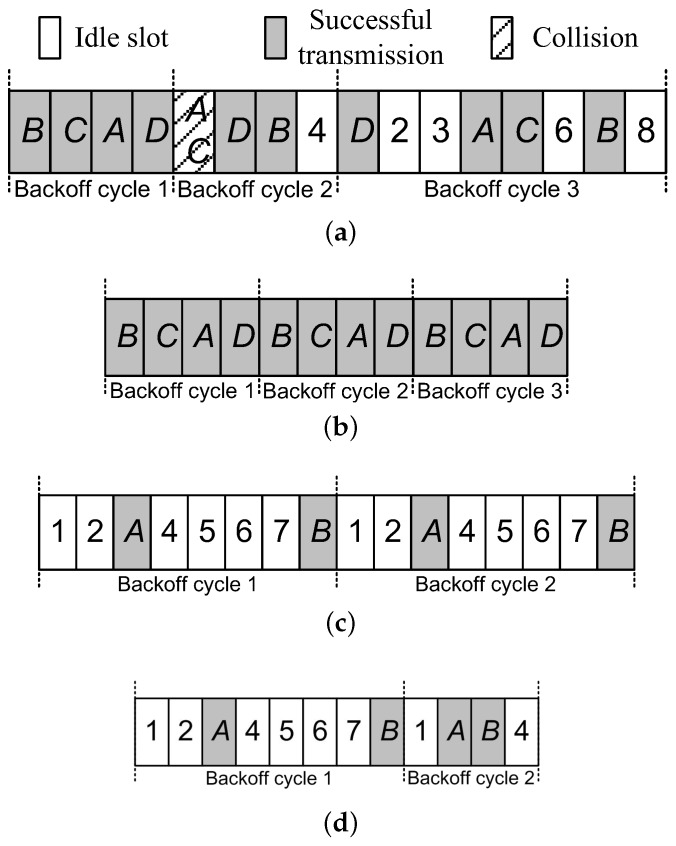
Channel accessing under different vehicle density. (**a**) Carrier Sense Multiple Access (CSMA)-based protocol under large vehicle density; (**b**) Our design under large vehicle density; (**c**) TDMA-based protocol under small vehicle density. (**d**) Our design under small vehicle density.

Our design synthesizes CSMA and TDMA by using two strategies in determining its next time slot to transmit a packet. Specifically, when the vehicle density is not large, all vehicles still randomly choose the next time slot. However, for the lager vehicle density, the time slot is reserved in an arbitral manner. The operation of our design can be illustrated in Figure 4.

We first compare our design with the CSMA-based protocol under large vehicle density. The principle of the CSMA-based protocol is shown in Figure 4a, where *A*∼*B* have packets to send. In the first backoff cycle, all vehicles send their packets at their respective time slot. In the second backoff cycle, however, *A* and *C* experience packet collision since all nodes set their backoff counters randomly in window [0, $CW_{min}$] as legacy 802.11 Distributed Coordination Function/Enhanced Distributed Channel Access (DCF/EDCA). Then, they randomly reset their backoff counters in a doubled contention window in the third backoff cycle. This example illustrates the shortcoming of the CSMA-based protocol under large vehicle density. In Figure 4b, to solve this problem, our design sets the backoff counters of all vehicles to a fixed common value (i.e., the number of vehicles) after successful transmissions. Therefore, all vehicles reserve the time slot in an arbitral manner and then avoid the collision again under the large vehicle density. Compared with the CSMA-based protocol, our design significantly increases the network goodput by 60%.

Under small vehicle density, our design utilizes the random channel-accessing strategy, which is much more effective than TDMA. Figure 4c shows the case of TDMA-based protocol when only *A* and *B* coexist. Since TDMA-based protocols always use the fixed frame length as the backoff cycle (i.e., eight in this example) regardless of whether or not the previous transmission is successful, many time slots are wasted due to the small vehicle density. Compared with the TDMA-based protocols, our design sets the size of contention window to the minimum value (i.e., 4) after successful transmissions in Figure 4d. Therefore, under small vehicle density, our design achieves a higher network goodput by 33% than TDMA-based protocols. In the following, we describe how to synthesize the CSMA- and TDMA-based protocol with only a few modifications of the legacy IEEE 802.11 backoff algorithm.

### 3.2. Backoff Algorithm

To avoid packet collisions, most CSMA-based MAC protocols use a simple yet effective random backoff algorithm, which is embedded in the IEEE 802.11 Distributed Coordination Function (DCF). The station always attempts to transmit its packet at an randomly chosen time slot. In our design, we only slightly modify the random backoff algorithm to introduce the resource reservation into the backoff procedure. Here, we use the number of vehicles in the transmission range *n* and its threshold $n^{*}$ to differentiate the vehicle density. Then, the vehicles adjust their channel accessing strategies based on the vehicle density.

We describe our proposed backoff algorithm in the following two cases.

- For the small vehicle density (i.e., $n\leq n^{*}$), the backoff time slot is randomly chosen to obtain high channel utilization. For each vehicle, if the previous transmission is successful, the next backoff time slot is uniformly chosen in [0, $CW_{min}$], where $CW_{min}$ is the minimum contention window size. If the previous transmission failed (i.e., the acknowledgment is not received), a retransmission is scheduled, and the value of CW is doubled at each retransmission up to a pre-determined value $CW_{max}$.
- For large vehicle density (i.e., $n>n^{*}$), the backoff procedure adopts the channel reservation at no extra cost. To prevent the vehicles from colliding with each other after a successful transmission, the number of time slots in subsequent backoff cycles is set to *n*, where *n* is the number of vehicles in the transmission range. Upon a failure of transmission due to hidden terminals, channel errors, or carrier sensing errors, the number of backoff time slots for retransmission is uniformly chosen in [0, *n*].

The pseudo-code of CTMAC is shown as Algorithm 1.

**Algorithm 1** The CTMAC Algorithm1: **if** the number of vehicles n is lager than $n^{*}$ **then**2:   **if** *the last transmission was successful* **then**3:    slot=n4:   **else**5:    slot=rand(0,n)6:   **end if**7: **else**8:   **if** *the last transmission was successful* **then**9:    $CW=CW_{min}$10:   **else**11:    $CW=min(2\times CW+1,CW_{max})$12:   **end if**13:   slot=rand(0,CW)14: **end if**


To synthesize the CSMA- and TDMA-based MAC protocols, we only add lines 1–7 into the legacy binary exponential backoff. These lines deal with the channel reservation with minimum modification to the existing implementation of IEEE 802.11p protocol. Therefore, our proposed algorithm can be readily applied, and is backward compatible with current protocols.

### 3.3. Implementation

We implement the design of CTMAC as adjusting its strategy according to network state. There are two key steps in our design. The first one is obtaining the number of vehicles *n* in the transmission range: the vehicle is required to maintain the realtime network state in an easy way with small overhead. In our design, we utilize the Beacon message as the notification. When receiving the Beacon message from its neighbour, the vehicle updates the number of nodes in its transmission range *n* according to the ID information in the Beacon message.

The second key step is setting the threshold value $n^{*}$. If $n^{*}$ is too large, the channel access will suffer more network collisions. In contrast, it will be too early to change to channel reservation, thus losing high flexibility and robustness. Here, we set the optimal value of $n^{*}$ as the number of vehicles when the network goodput of CSMA- and TDMA-based protocols are equal in Equations (11) and (12). Finally, we calculate the optimal value of threshold $n^{*}$ as

(13) $$n^{*}=\left\lfloor \frac{\log((s+D+1)/(N\times p+s+D))}{\log(1-p)}\right\rfloor .$$

## 4. Performance Evaluation

In this section, we compare the performances of CTMAC with VeMAC and IEEE 802.11p by using NS2 simulator [9]. The simulation settings are shown in Table 1. As mentioned before, the IEEE 802.11p protocol employs a random backoff scheme. We first evaluate the basic performance under the impact of vehicle density, and then analyze the protocol performances in two VANET applications: online game and safety warning. In the simulations, each test is repeated 10 times. The average values of the test results are calculated with 95% confidence intervals. In the following, we give the performance metrics, simulation setup, and performance analysis.

### 4.1. Performance Metrics

*Number of Collision Events* is the metric to measure the channel contention, and can be generated when several neighboring nodes decide to access and transmit their packets at the same time. When the number of collision events is small, high channel utilization is obtained.

*Delay* is defined as the average time from the moment when a vehicle starts trying to send the packet until the end of its successful transmission, which is a crucial factor in time-critical safety applications [12]. The faster the safety message propagates, the more efficiency the corresponding protocol achieves in terms of satisfying the urgent delay requirement of emergency application.

*Network Goodput* is defined as the fraction of the channel capacity used for data transmission. The goal of an efficient MAC protocol is to maximize the throughput while minimizing the delay for user-oriented applications.

### 4.2. Basic Performance

#### 4.2.1. Impact of Vehicle Density

We use a dynamic scenario to evaluate the basic performances under different vehicle densities. We model a straight 6400 m highway scenario where vehicles are moving on a two-way highway at different speeds. The number of nodes varies from 160 to 640, and the ratio of static nodes to motion nodes is 1:3. The average speed of vehicles’ motion is 100 km/h. In order to simulate the communication traffic, we assume each node sends data packets with 200 B size to their neighbours with the transmission rate of 100 Mbps and the transmission range of 400 m. The time slot is 50 μs, and the frame length of VeMAC is set as 100.

**Figure 5:**
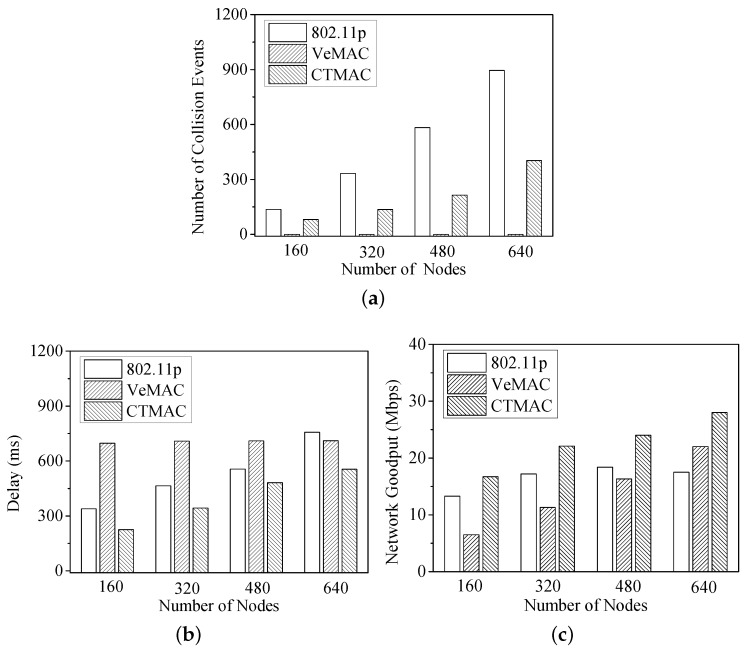
(**a**) Number of collision events; (**b**) delay; and (**c**) network goodput under different vehicle densities.

Figure 5a shows that for both IEEE 802.11p and CTMAC, the number of collision events becomes larger as the number of vehicles increases. When the number of vehicles is 160, almost all vehicles in the network can be accommodated, and the collision rate is extremely low. On the other hand, when they cannot be accommodated, the number of collisions rises considerably, especially for the 802.11p protocol. Compared with the other two protocols, VeMAC achieves a zero collision rate throughout the test due to its channel reservation.

Figure 5b shows the delay under different vehicle densities. Since it uses a fixed frame length as the backoff cycle, VeMAC provides a predictable bounded delay no matter how large the number of vehicles. IEEE 802.11p gets a smaller delay under light channel contention, but a lager delay when contention becomes heavy. Because it adaptively shifts the channel accessing manner according to the network state, CTMAC protocol obtains the smallest delay.

In Figure 5c, it is shown that the network goodput of CTMAC is the highest among the three protocols. When the number of vehicles is 640, the network goodput of CTMAC is about 45% and 21% higher than that of 802.11p and VeMAC, respectively. This result is explained by the fact that CTMAC adjusts the channel accessing according to the vehicle density. When the vehicle density increases, CTMAC reduces the randomness in channel accessing and effectively alleviates the packet collision. On the other hand, in a lightly loaded network, CTMAC reverts to legacy random accessing and achieves high channel utilization.

#### 4.2.2. Impact of Vehicle Speed

In this section, we change the vehicle speed to evaluate the protocol performances. We set the number of vehicles as 400 and vary the average vehicle speed from 50 km/h to 125 km/h. The other simulation settings are the same as those in Section 4.2.1.

**Figure 6:**
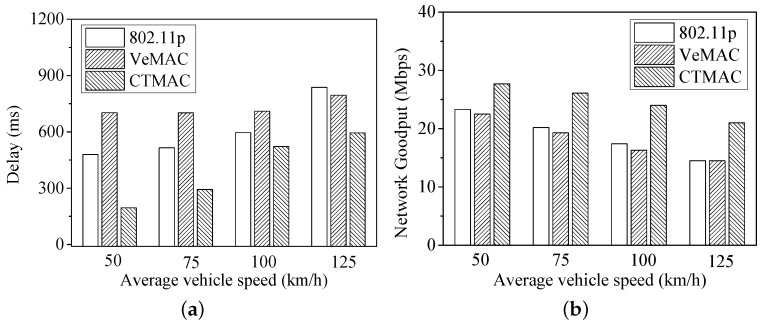
(**a**) Delay; and (**b**) network goodput under different vehicle speeds.

Figure 6 shows the delay and network goodput under different vehicle speed. When the vehicle speed increases, the network topology becomes more dynamic, resulting in more channel conflict and idle time slots. Therefore, for all protocols, the delay is increasing while the network goodput is reduced. Nonetheless, CTMAC obtains the best performances among the three protocols.

#### 4.2.3. Impact of Transmission Range

In this test, we evaluate the protocol performances with different transmission ranges. The number of vehicles is 400, and the transmission range is changed from 300 m to 500 m. The other simulation settings are the same as those in Section 4.2.1.

**Figure 7:**
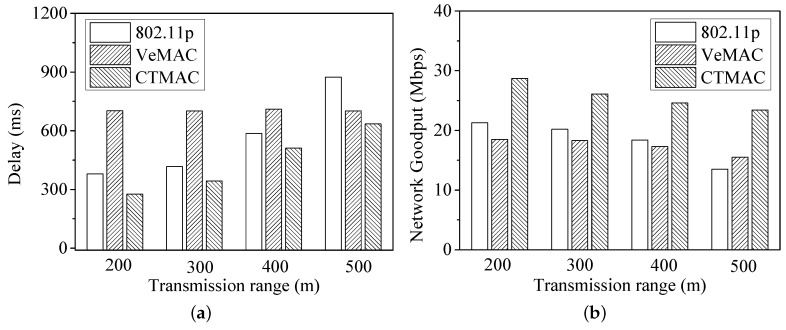
(**a**) Delay; and (**b**) network goodput under different transmission ranges.

Figure 7 shows the delay and network goodput under different transmission ranges. When the transmission ranges become larger, more vehicles are involved in the same transmission range, leading to a higher likelihood of packet collision. Due to high packet collision under the large transmission range (i.e., 500 m), IEEE 802.11p’s delay is larger than that of VeMAC and CTMAC. Meanwhile, for VeMAC, the number of idle time slots increases with larger transmission range, thus degrading the network goodput. Compared with the other protocols, CTMAC achieves a smaller delay and larger network goodput.

### 4.3. Online Game Application

The online game is one of the most popular applications in VANET. Each player periodically generates multihop transmissions to the other players in the online game application. The generation rate is a very important factor for the game quality and player experience. Thus, we vary the transmission interval to test the performances under different congestion states of the wireless channel.

In this test, 500 vehicles with the average speed of 100 km/h are randomly moving on 5 km four-lane highway. We randomly select 50 vehicles as the players, which periodically generate 200 Byte sized packets to the other players. We evaluate the delay performances considering different generation intervals with each player. Specifically, the packets are generated at each vehicle every 10, 50, or 100 ms.

**Figure 8:**
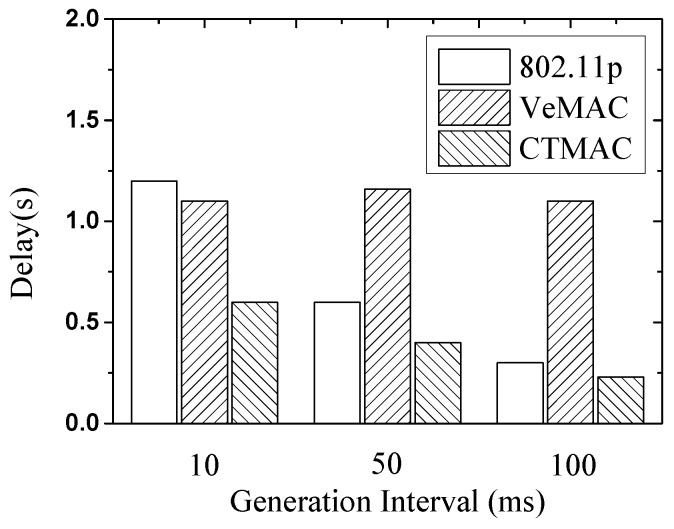
Online game: delay with different generation interval.

We measure the average value of game event delay, which is defined from time that the player sends packet to the time that all the other players receive the packet. Figure 8 presents the delay results with different generation intervals. It could be observed that when the generation rate is 10 ms, the game event delay of 802.11p are larger than 1.2 s. This result is attributed to the high packet collision. If the generation interval is 50 ms or 100 ms, the game event delay drops. As expected, in the three protocols, CTMAC gets the lowest delay with all different generation intervals.

### 4.4. Safety Warning Application

Acident detection and avoidance by the dissemination of safety messages is considered as one of the most important services of VANETs. When an accident happens, the safety warning message should be broadcasted through the following vehicles (i.e., platoon). In the test of safety warning application, we modeled a car platoon of 30 cars traveling at 100 km/h with an inter-vehicular distance of 25 m. Here, we should note that the inter-vehicular distance is set as a fixed value to compare the test results of speed difference and the distance between two consecutive vehicles after stopping. Only the first vehicle is chosen as the emergency message source, which broadcasts the emergency message backward to all the following vehicles along the highway.

At a certain point, the first vehicle in front of the platoon has an accident, and its speed is quickly reduced with a deceleration of 8 m/s${}^{2}$. The reaction time of drivers is uniformly distributed between 0.75 and 1.5 s. Drivers are assumed to brake after the reaction time if their vehicle receives a warning message, or if one of the two vehicles in front of them brakes. After braking, the vehicles decelerate at 8 m/s${}^{2}$ until they stop.

**Figure 9:**
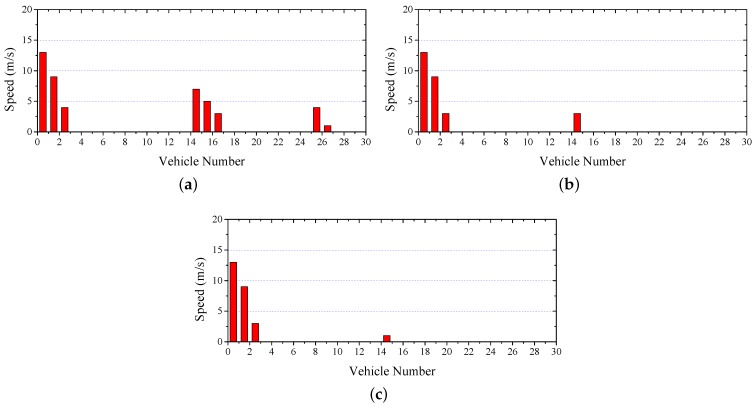
Safety Warning: Speed difference between two consecutive vehicles. (**a**) IEEE 802.11p; (**b**) VeMAC; (**c**) CTMAC.

Figure 9 shows the speed difference between two consecutive vehicles after stopping. An accident occurred when the speed difference was not zero. Since the wireless transmission range is limited (about 300 m), the vehicles have to relay the safety warning messages by multihop broadcasting. Therefore, if the transmission delay of each hop is small, the safety warning messages could be received by the following vehicles quickly. Since adopting channel reservation under heavy contention, both VeMAC and CTMAC obtain low delay and thus effectively reduce the number of vehicles involved in a chain accident. When using CTMAC, vehicle 14 hits the preceding vehicle at a slightly lower speed than VeMAC.

**Figure 10:**
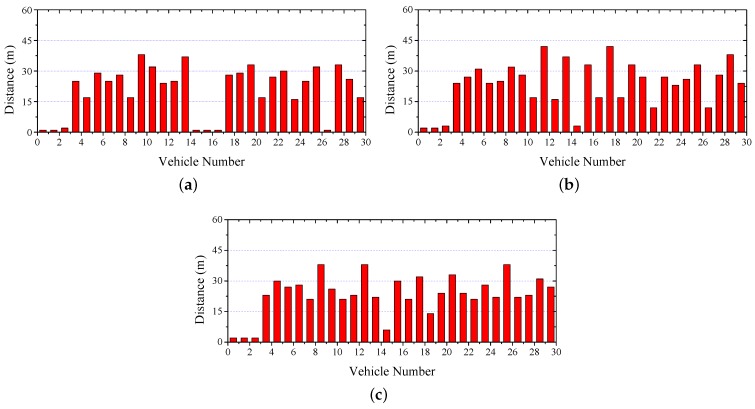
Safety Warning: Distance between two consecutive vehicles. (**a**) IEEE 802.11p; (**b**) VeMAC; (**c**) CTMAC.

Figure 10 shows the distance between two consecutive vehicles after stopping. If the distance is less than 4 m, an accident is assumed to happen. It is shown that—since it has the lowest transmission delay—the average distance in CTMAC is smaller than the other two protocols.

**Figure 11:**
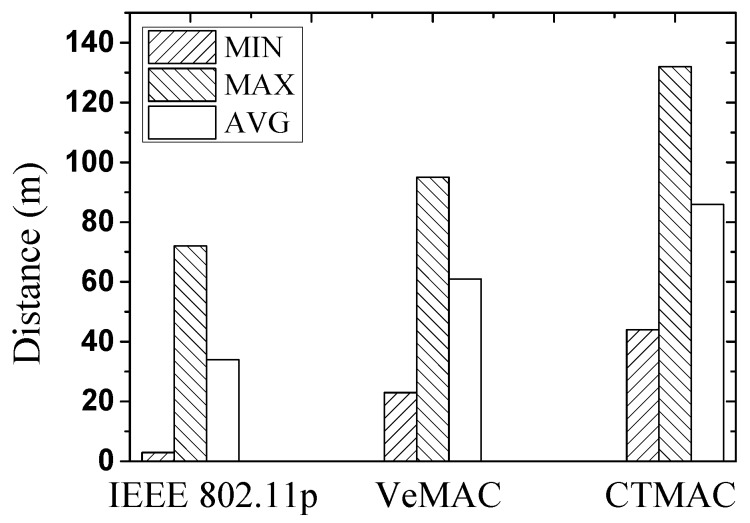
The minimum, maximum, and average distance between two consecutive vehicles.

To evaluate the protocol performance in more realistic scenarios, we set 500 vehicles moving on 5 km of a four-lane highway. The vehicle speed and initial inter-distance are uniformly distributed as (80 km/h, 120 km/h) and (50 m, 150 m), respectively. Figure 11 shows the minimum, maximum, and average distance between two consecutive vehicles after stopping compared. It is shown that CTMAC obtains the largest distance between two consecutive vehicles after stopping, effectively helping more vehicles to avoid a crash.

### 4.5. Urban Scenario

**Figure 12:**
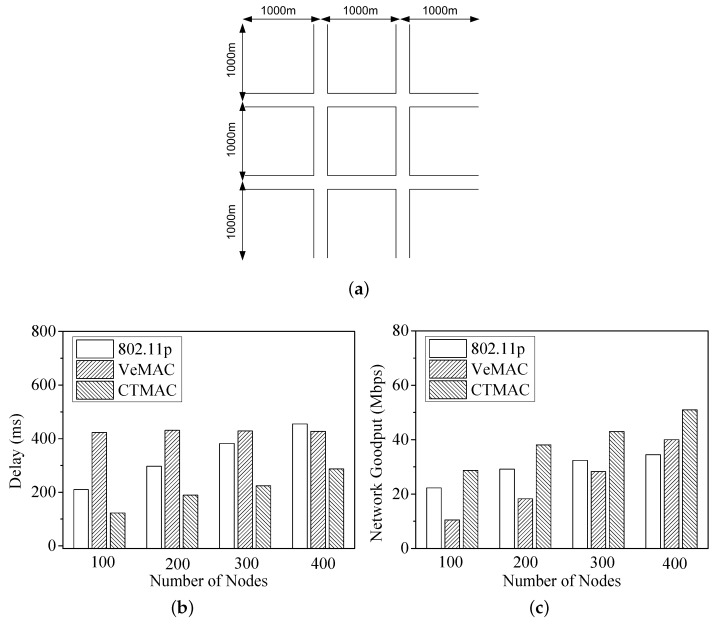
Urban Scenario: Delay and network goodput under different vehicle densities. (**a**) Urban Scenario: 3000 m × 3000 m; (**b**) Delay; (**c**) Network goodput.

The second test scenario is urban, consisting of four intersections, as shown in Figure 12a. Each road segment contains two bidirectional lanes. Five hundred vehicle nodes move at a random speed between 30 km/h and 60 km/h. Each vehicle node sends 200 B data packets to their neighbours with 100 Mpbs transmission rate and 400 m transmission range. The time slot is 50 μs, and the frame length of VeMAC is set as 50.

Figure 12b,c shows the delay and network goodput under different vehicle densities in the urban scenario. With the increasing of number of vehicles, the delay and network goodput also increase. Compared with IEEE 802.11p and VeMAC, CTMAC obtains a smaller delay and larger network goodput due to its synthesis of the random accessing channel and arbitral reserving channel together.

## 5. Related Work

Generally, MAC protocols fall into one of two broad categories: CSMA-based and TDMA-based. The CSMA-based protocols do not require any predefined schedule; each node will compete for channel access when it needs to transmit. As the standard deployed to enable vehicular communication, IEEE 802.11p protocol uses a priority-based access scheme that employs both Enhanced Distributed Channel Access (EDCA) and Carrier Sense Multiple Access with Collision Avoidance (CSMA/CA) mechanisms. However, when several neighboring nodes sense a free channel and decide to access and transmit their packets at the same time, packet collisions happen. Therefore, the IEEE 802.11p protocol does not provide a reliable broadcast mechanism with bounded communication delay.

As an enhanced CSMA-based protocol, Receiver Consensus (ReC) [13] uses geographical information to reduce the packet collision and transmission delay. In ReC, the selected receiver is the nearest vehicle to the centroid of neighboring vehicles that have not received the packet. Upon receiving the packet, the selected receiver retransmits immediately. Thus, the packet collision is reduced, while at the same time the receiver can retransmit the packet to the following vehicles without delay. However, due to the vehicle’s high mobility, there exists a great practical difficulty—ReC requires a complete and continuously updated knowledge of the neighboring vehicles.

To avoid inaccurate position estimation in a highly mobile environment, Privileged Inter Vehicle Communication Architecture, (PIVCA) [14] utilizes Hello messages to get the realtime position information of other vehicles around. With the neighbour position information, PIVCA prioritizes farther vehicles in forwarding packets. In particular, the broadcast delay is computed based on the contention window (CW) in IEEE 802.11p MAC protocols. The farther vehicle has a smaller CW, and thus will have lower waiting delay before relaying the message.

Another emerging area of research in the field of VANETs is TDMA-based MAC protocols. To avoid packet collision, the time is divided into slots, and only one vehicle can access the channel at each time slot in TDMA-based protocols. Since all vehicles use the same channel at different times, the disadvantages of the IEEE 802.11p standard is eliminated, and the time to access the channel is reduced when node density is high. However, many issues arise due to the high vehicle mobility, degrading the performances of these TDMA-based MAC protocols [15,16,17].

Several TDMA-based MAC protocols are proposed to address the problem in mobility scenarios. In AdHoc MAC [18], the vehicles are grouped into a set of clusters without centralized coordination. To avoid the hidden terminal problem, the vehicles broadcast the status of time slots used by all the other vehicles within its one-hop neighborhood. Upon receiving the status information, each vehicle knows the time slots used by all of the vehicles within its two-hop neighborhood and the set of accessible time slots. Then, the vehicles choose the free time slot to transmit its data, without causing any packet collisions.

VeMAC [11,19] is a multi-channel TDMA-based MAC protocol proposed for VANETs. In the control channel, the vehicle exchanges the status information of time slots with its one-hop neighbours. Then, each vehicle determines its time slot in the service channel and achieves contention-free accessing channel. However, though communications over the service channels has no overhead, the overhead of the control channel is a considerable issue.

Unified TDMA-based Scheduling Protocol (UTSP) [20] uses roadside units to collect the information, including the channel state, moving speed, and Access Category of the vehicles within its communication range, and then assigns the time slots to the vehicles. UTSP achieves high network throughput and ensures fairness between vehicles; however, it was designed to support only VANET with the roadside units.

Cooperative AdHoc MAC (CAH-MAC) [21] protocol is a cooperative TDMA-based MAC protocol in VANET with the aim of improving network throughput in poor channel. Upon a transmission failure, a neighboring vehicle called *helper* offers cooperation to relay the failed packet during an idle time slot. The shortcoming of CAH-MAC is that the helper vehicle is not able to transmit its own data when it retransmits the failed packet. Furthermore, when the free time slot is used for retransmission, the number of time slots available for newly joining vehicles is reduced.

CSMA-based protocols do not require any predefined schedule, and achieve high goodput for low vehicle density. Since there is no guarantee of successful transmission, however, CSMA-based protocols may cause problems such as packet loss and large delay for large vehicle density. TDMA-based MAC protocols work better in improving transmission reliability and reducing the delay when the vehicle density is large. However for low vehicle density, the fixed frame length increases the wasted time in idle slots, and eventually results in lower goodput.

In contrast with the enhanced CSMA and TDMA-based MAC protocols, our solution CTMAC tackles the channel accessing problem through a different perspective: we adjust the contention window by dynamically synthesizing CSMA- and TDMA-based MAC Protocols. The key difference is that existing solutions tackle the channel accessing problem with either random backoff or resource reservation, while our solution introduces resource reservation into the backoff procedure to address the dynamic scenarios with both small and large numbers of vehicles, where existing solutions become ineffective.

Similar to our design, a semi-random backoff method (SRB) is proposed in wireless LANs [22]. Upon a successful packet transmission, the wireless station to set its backoff counter to a deterministic value. In the case of failed transmission, the station will revert to the standard random backoff method and probe for a new available time slot. Though SRB obtains higher network efficiency, it is only suitable for the static wireless LANs, not for dynamic VANETs with underlying mobility and topology changes. Specifically, SRB always sets the backoff counter as a fixed value under different station density. When the vehicles join and leave, the packet collision or channel wastage will easily happen, degrading the network performance. In this case, our solution sets the backoff counter according to the vehicle density, seamlessly switching the channel accessing strategy between CSMA and TDMA in a flexible and robust manner without causing goodput loss.

## 6. Conclusions

This work presents CTMAC for highly dynamic scenarios in VANETs. We synthesized the random backoff and resource reservation to address the channel accessing issue. CTMAC only adjusts the contention window, which avoids extra overhead. Our design can obtain significant goodput gains in dynamic scenarios with different vehicle density. We evaluate the performance of CTMAC with at-scale tests. The test results indicate that with CTMAC, we effectively reduce the number of packet collision events and accessing delay, hence improving the network goodput by 45%.

## Figures and Tables

**Table 1:** Road Traffic Parameters and medium access control (MAC) protocol Setting. TDMA: Time Division Multiple Access. DIFS: Distributed Inter-frame Spacing.

	Parameter and Setting	Value	Parameter and Setting	Value
	Road area	600 m× 30 m	$CW_{min}$	15 slots
	Transmission range *R*	300 m	$CW_{max}$	1023 slots
	Channel Propagation	TwoRayGround	Number of slots *N* in TDMA Frame	50 slots
	Time slot *σ*	20 μs	Wireless transmission rate *r*	100 Mbps
	DIFS time *D*	50 μs	Packet size *s*	1000 Byte
